# Deus Ex Machina: An Ethnographic Exploration of Technology, Death and Decision‐Making in Respiratory Care

**DOI:** 10.1111/1467-9566.70091

**Published:** 2025-09-28

**Authors:** David Wenzel, Jennifer Creese, Eleanor Wilson, Michael Jones, Christina Faull

**Affiliations:** ^1^ Department of Population Health Sciences University of Leicester Leicester UK; ^2^ School of Health Sciences University of Nottingham Nottingham UK; ^3^ Respiratory Department University Hospitals of Leicester Leicester UK; ^4^ LOROS Hospice Leicester UK

**Keywords:** end‐of‐life care, ethnography, Foucault, power/knowledge, respiratory care

## Abstract

Noninvasive advanced respiratory support (NARS) is widely used in acute respiratory failure, including at the end of life, where its use is ethically and emotionally fraught. This ethnographic study examines how decisions to initiate, sustain or withdraw NARS are negotiated within the institutional and moral complexities of a UK hospital. Drawing on fieldwork including nonparticipant observation, reflective fieldnotes and composite narratives, this paper explores how dying is not simply recognised but discursively produced through clinical interactions. Guided by Foucault's concept of power/knowledge and Orlikowski's theory of technology‐in‐practice, this study shows how authority circulates unevenly across professional boundaries and how machines become nonhuman actors that structure time, command clinical attention and produce epistemically privileged outputs. These outputs, especially blood gas samples, change the flow of power/knowledge and can override patient experience, shaping decisions around palliation. By tracing how clinical truth is co‐produced through medical technology, discursive hierarchies and institutional logics, this paper intervenes in sociological debates on end‐of‐life care, technological agency and the moral labour of frontline staff. It argues that recognising dying is not merely a clinical judgement but an emergent, distributed achievement, one shaped as much by machines and metrics as by human actors.

## 1 Noninvasive Advanced Respiratory Support

Noninvasive advanced respiratory support (NARS)—including continuous positive airway pressure (CPAP) and bilevel airway pressure (also known as noninvasive ventilation or NIV)—is used in the treatment of respiratory failure (Pierson 2009). Both treatments involve the delivery of pressurised air and oxygen, most often via a tight‐fitting mask interface. Patients treated with NARS are seriously ill, and mortality rates among those receiving this treatment are high. The single largest patient group receiving NARS is patients with chronic obstructive pulmonary disease (COPD), where the inpatient mortality rate is 12% (Osadnik et al. 2017). For those aged over 65 suffering from COVID‐19 pneumonitis, the mortality rate can be as high as 85% (Aung et al. 2021).

The intervention is burdensome: NARS can produce feelings of claustrophobia, anxiety, breathlessness and fear (Smith et al. 2015) but can also act as a symbol of hope for patients and a chance for recovery (Christensen et al. 2017). Staff recognise the duality of this role—balancing the burden of treatment against the chance of recovery—but it is also associated with significant moral injury, where clinicians feel they have acted in contravention of their conscience. Within NARS care, this may take multiple forms, ranging from guilt about compelling a dying patient to endure burdensome treatment to a sense of complicity in death when withdrawing therapy judged to be ineffective (Wenzel et al. 2022). The decision‐making around these patients is complex and requires input from the entire multidisciplinary team, but the role of the machines themselves in the decision‐making process is understudied.

Treatment with NARS necessitates intensive, active clinical monitoring. Although routine observations provide important contextual information, blood gas sampling offers data that cannot be obtained noninvasively (Davidson et al. 2016). Typically, acutely unwell patients undergo arterial sampling from the radial artery at the wrist, which reveals oxygen levels, carbon dioxide and blood pH. Elevated carbon dioxide with an acidic pH, or low oxygen, may indicate the need for NARS. Once commenced, treatment is monitored through repeated blood gases to guide settings and assess response. For stable or recovering patients, less invasive capillary samples from the ear may be used, though in patients with poor peripheral perfusion, arterial sampling remains necessary. These results are printed onto small scrolls of thermal paper at near‐patient testing sites. They are simple to understand but complex to master, and the changes made to the machine as a result are a specialist area of care.

In this paper, we bring together Foucauldian power/knowledge and Orlikowski's notion of technology‐in‐practice to examine how authority over dying is not only hierarchically distributed but also technologically mediated. This raises new questions about the epistemic politics of end‐of‐life care and how novel technology shapes the sociological understanding of death.

## 2 Conceptualising Dying, Decision‐Making and Technological Authority

Contemporary dying in Western healthcare settings is deeply shaped by clinical expertise and institutional routines. Timmermans (2005) summarised this influence as ‘death brokering’ and described the process through which medical experts make death culturally appropriate. The role of technology in brokering culturally appropriate death is described, and the risk of technocratic death is explored (where death is drawn out using advanced technology (Moller 2000)).

Although Timmermans acknowledges the presence of medical technology in these processes, less attention has been paid to how technology itself, and the professional expertise it necessitates, actively shape the conditions under which death is recognised, deferred or denied. He observes that ‘a consensus among healthcare providers is only necessary to withdraw or withhold care; as long as at least one key member of the medical team wants to continue with therapy, the treatment paradigm will prevail’ (2005). However, who counts as a ‘key’ member is not fixed. The authority to declare a patient as dying and thus to broker a shift towards palliative care, is relational, contingent and institutionally unstable.

These dynamics are particularly pronounced in the use of NARS, a technology frequently employed for patients with chronic illnesses such as COPD or obesity hypoventilation syndrome. These conditions are marked by fluctuating trajectories, where patients may survive one exacerbation only to decline during another (Lunney et al. 2002). The stakes of withdrawal are high: Withdrawing NARS too early risks precipitating avoidable death, whereas continuing it too long may prolong suffering. This uncertainty reinforces institutional norms favouring continued intervention, and thus, the ability to declare treatment as futile becomes a matter of authority, one shaped by professional role, institutional alignment and the outputs of the machine itself.

This has a significant impact on the realities of end‐of‐life care planning, where patients' choices become limited and driven by reaction to technological and biological imperatives. This widens the already significant mismatch between policymakers of choice and planning (Borgstrom and Walter 2015) and the discursive turbulence of NARS at the end of life. Institutional documents guiding NARS withdrawal weigh the presence of advance care plans heavily in clinical decision‐making (Wenzel et al. 2024), yet in practice, few patients complete such plans for these situations. The origins of this gap are best summarised by Borgstrom (2014) in an ethnographic exploration of end‐of‐life care when discussing this issue with a respiratory nurse, ‘the uncertainty of the conditions her patients suffered from made advance care planning impractical and optimistic… scenarios of unpredictable hospitalisation and sudden death are difficult to accommodate within this framework’ (p. 150).

In this paper, we build on these insights by examining how NARS functions not merely as a life‐sustaining device, but as a technology that participates in the brokering of death. Drawing on Foucault's (1980) theory of power/knowledge and Orlikowski's (2000) concept of technology‐in‐practice, we align with a broader tradition in medical sociology to explore technologies’ effect on care not as neutral tools, but as socially and ethically consequential actors (Casper and Morrison 2010). We explore how the NARS machines structure decision‐making, mediate professional hierarchies, and co‐author the social and institutional meanings of dying within acute hospital settings.

## 3 Foucauldian Power/Knowledge

To interrogate how the authority to broker death is exercised in the context of medical uncertainty around NARS, it is necessary to consider not only who holds the authority to withdraw care, but also how that authority is legitimised, challenged and enacted. In practice, this authority circulates among members of the healthcare team, operating across different times, spaces, roles and levels of seniority, each of whom may shape the trajectory of a patient's care (Anderson et al. 2021).

We draw on Foucault's (1980) conceptualisation of power/knowledge to explore how the authority to change and shape the patient's management plan is dynamically altered by specialist knowledge and the power to declare ‘truth’.

Foucault wrote extensively on the concept of how knowledge becomes accepted as ‘fact’ and the influence of power in determining what discourses are valid and accepted and which are dismissed (Foucault 1980, 1982). In relation to NARS care, the greatest impact of this concept is the ability to declare a patient is ‘dying’ rather than critically ill. The declaration of dying and an adoption of a palliative approach to treatment require power that is intricately linked to knowledge.

Foucault did not deny individual expertise but emphasised that what is accepted as valid or authoritative knowledge depends less on inherent genius and more on the interplay of institutional power, discourse and accepted norms (Mills 2003). In this context, he rejects the idea that the most knowledgeable member of the team (likely a senior respiratory doctor) can simply recognise death and futility most accurately. Instead, Foucault relied on the concept of power/knowledge to explain the deeply intertwined links between knowledge and power.

Power, within this framework of understanding, is not a possession that allows powerful agents to exact their will over powerless agents. Rather, power is performed, executed more as a strategy than wielded as an object, ‘Power must be analysed as something which circulates, or as something which only functions in the form of a chain… individuals are the vehicles of power, not its points of application’ (Foucault 1980, 80). Thus, the authority to declare a patient as dying is not simply granted by institutional hierarchy; it is constructed and negotiated through discourse, institutional norms and the interplay of power/knowledge embedded in everyday clinical practice.

Although senior doctors derive significant authority from institutional norms, this framework also allows room for subject expertise and clinical discourse to shape the decision to declare a patient as dying. This discourse may be provided by specialist and nonspecialist members of the healthcare team as well as senior and junior doctors.

Through this lens, we can see that decisions in care may be less about the isolated clinical judgement of an individual and more about the product of negotiated truths shaped by institutional discourse, historical practice and the interplay of expertise. The authority to shift a patient's care from active treatment to palliation does not arise in a vacuum; it emerges through a network of interactions, note entries, handovers and multidisciplinary discussions. These are key sites where power circulates, and knowledge is accepted or challenged—the patient's trajectory is gradually redefined through discourse. The focus of this ethnographic study is to explore which voices are more readily heard and which may be sidelined—exploring clinical decisions not only as medical acts but also as discursive performances.

## 4 Technology‐In‐Practice

To understand the role of the NARS machines in shaping care decisions, it is essential to move beyond viewing them as neutral tools or background instruments—objects that do not wield power. Instead, we draw on the concept of technology‐in‐practice (Orlikowski 2000) to explore how technologies are enacted through human practice and, in turn, help structure those very practices. In this framework, technologies are not static artefacts but are performed into being through use. They shape and are shaped by the routines, values and institutional structures within which they are embedded.

The NARS machine does not simply deliver air and oxygen; it structures time, mandates action and produces data that reorientate clinical focus. Its presence generates workflows: cycles of blood gas monitoring, changing delivery pressures and optimising mask fit. This aligns with Orlikowski's argument that technologies ‘enact particular structures’ in daily practice, creating *technologies‐in‐practice*—the emergent, patterned use of tools shaped by organisational norms and actor intentions (Orlikowski 2000).

In this context, NARS also becomes a nonhuman actor, a term drawn from actor‐network theory (Latour 2005), which suggests that objects can hold agency within sociotechnical networks. The machine ‘acts’ by prompting sedation for compliance, influencing treatment continuation and participating in truth production (providing evidence of a patient's recovery or lack thereof). The machine thus becomes more than a device; it is a co‐author of clinical decisions.

The authority of the machine is further reinforced by its ability to produce quantifiable outputs (gas values, mask compliance measures, volumes of gas being delivered) that are interpreted as evidence of treatment efficacy or futility. These outputs are afforded epistemic weight and serve to mediate communication not only among clinical teams but also between clinicians and families. There is no objective or quantified output for patient comfort, which can contribute to a prioritisation of survival over comfort and compliance over autonomy.

Interpreting the data from NARS machines is complex and requires specialist knowledge. This means the creation of meaning from the data may vary by user group (specialist vs. nonspecialist clinicians). This creates a potential variance in the behaviour of the machine within the actor‐network, shaped not by the machine itself but by the differing professional cultures, training and values of those who interact with it.

In sum, the NARS machine is not a passive instrument but an active element in the clinical ecosystem. It structures interactions, commands attention and helps produce clinical ‘truths’. Understanding technology‐in‐practice allows us to see how clinical decisions, patient trajectories and experiences of dying are mediated not only by people and discourse, but also by the machine itself and the practices it enforces.

## 5 Research Design and Methods

To explore clinical decisions and the care provided by interlocutors, author D.W. undertook ethnographic fieldwork in a single, large, acute National Health Service (NHS) trust in England, UK, between November 2024 and February 2025. D.W., a clinical academic, shadowed multiple healthcare workers with different multidisciplinary roles across a full spectrum of day, night and weekend shifts. D.W. is a palliative care speciality trainee, and he has previously worked across all areas of the field in a clinical capacity.

Fieldnotes were generated contemporaneously with paper and pen or digital voice notes. All fieldnotes contained a section of reflexive commentary that included consideration of how positionality may have shaped observations. These notes, and the interpretation of data, were discussed closely with supervisors, especially J.C.—an experienced ethnographer.

Positionality was most often partially disclosed to interlocutors, with D.W. introducing himself as a PhD student wearing a student lanyard. On many occasions, D.W. was known to the clinical team in other capacities but always as a member of a different clinical team. D.W.'s positionality and disclosure thereof are best described as ‘living in the hyphen’ as described by Dwyer and Buckle (2009). D.W. entered the field wearing scrubs and a student lanyard but did not attempt to conceal his clinical background if directly asked.

In this way, he was able to present himself as an insider (clinically trained) while remaining an outsider (introduced as a student, not a member of the clinical team). This hyphen presentation allowed for exploration of interlocutor views without making junior team members feel unduly ‘examined’ and to limit the impact of power imbalance on interlocutor behaviour.

D.W. attended wards with patients receiving NARS and consented them to observe care using encrypted audio‐recorded verbal consent or a consultee declaration process. This study was approved by an NHS Research Ethics Committee, Wales REC7, on 2 August 2024 (IRAS ID 339785, REC reference 24/WA/0215) and given local green light by the University of Leicester as well as the trust's local research and innovation department.

Fieldnotes made with pen and paper during placement were typed up in full within 24 h of the fieldwork's conclusion in Microsoft 365. All notes were anonymised at the point of collection. To ensure complete anonymity for patients, location was kept to ward level, no ages were recorded, and rarer disease types were anonymised to ‘respiratory illness’.

Data were interpreted iteratively throughout the study utilising reflexive thematic analysis (Braun and Clarke 2023); themes are linked through interpretative meaning. Navigation within the field was aided by this contemporaneous analysis, where areas of care that needed further exploration were targeted specifically within the field.

In total, 108 h of direct observation of care and care activities were conducted. Fourteen patient journeys were observed, with almost half followed from the initiation of therapy through to the patient's death.

Illustrations of interpretative themes were created through the use of composite narratives. This format of data representation was chosen to allow empathetic illustration of the data (Bleakley 2005) and to ensure interlocutor anonymity (Sajadi et al. 2023). Composites were constructed using the Johnston (2024) six‐step methodology—whereby composites are developed in two halves, supported by quotes and extracts from data. Titling of the composites is represented by the title of the theme, and all included names are pseudonyms.

The endpoint of the study was agreed between D.W. and supervisors when sufficient information power to answer the primary study aim had been collected (Malterud et al. 2016). The primary study aim was ‘to explore the experiences of care for end‐of‐life for patients using, or having recently used, NARS for the treatment of critical illness’ with specific objectives to explore ‘how staff discuss and communicate that patients starting NARS are critically ill’ and to ‘explore the decision‐making process that determines when a patient's NARS is considered clinically to not be working and death is inevitable’. Findings from the study are presented below, each with an illustrative composite narrative.

## 6 Fractured Authority, Uncertain Autonomy

‘It’s flu pneumonitis, David, not one for you,’ said Vidhya, the consultant, firmly ruling out NARS for Nirasha, a woman in her late 60s with severe respiratory failure and significant learning needs, whose family never left her side. Yet the next morning, I found a mask strapped to Nirasha’s face: the night registrar, Alex, had started NARS under pressure from the family, uncertain and alone in the absence of senior support. At board round (in Alex’s absence), Vidhya publicly chastised the decision, lamenting the incapacity of juniors to manage complex cases, but offered no alternative for the out‐of‐hours reality. That night, Alex reviewed Nirasha again as her condition deteriorated, increasing pressures despite knowing the plan, ‘I’d rather be criticised for doing something than for doing nothing.’ Nirasha died the following morning with the mask still in place; the decision may have been flawed and forbidden, but at least Nirasha died on someone else’s shift.

The technical management of NARS is complex and demands specialist knowledge. Although registrars like Alex may have considerable clinical experience, they often operate without the deep domain expertise of consultants. As such, they inhabit a liminal epistemic space, trusted for their medical training but not yet transformed to consultancy and institutional authority. Foucault describes that ‘it is not possible for power to be exercised without knowledge’ (Foucault 1980), yet institutional structures paradoxically expect registrars to make high‐stakes decisions overnight, often without direct senior input. These decisions (such as initiating or escalating NARS) become vulnerable to retrospective scrutiny not because they are clinically irrational, but because they lack the discursive legitimacy granted by institutional authority during daylight hours.

During ethnographic placement, there was evidence of clinically irrational decisions being made; for example, a registrar, Lewis, declined to treat a patient's developing acidosis due to an erroneous perception in the guidelines about when to start NARS or not. However, not all of the criticised decisions made by junior staff were irrational.

The rise of evidence‐based medicine (EBM) promised to democratise clinical decision‐making, suggesting that access to data would flatten hierarchies and empower juniors to act autonomously (Grimes 1995). However, as Timmermans and Berg (2003) explored, junior doctors are ‘at the bottom of a steep authority ladder’ (p. 159), and this egalitarian vision fails to account for the technical and hierarchical realities of clinical work. Junior doctors may know the guidelines and grasp clinical uncertainty well, but their interpretations of evidence are often devalued unless endorsed by more senior or specialised actors.

Alex could have phoned a more senior doctor to discuss the deterioration, but even that decision was shaped by tacit institutional norms. *‘The consultant on isn't a ventilation specialist anyway, so they won't want to change the plan,’ Alex explained*. Here, the circulation of power/knowledge around respiratory subspecialists was so dominant that even consultants outside this epistemic network were perceived as lacking legitimate authority. Moreover, Alex feared being labelled ‘acopic’—a colloquialism for doctors deemed ‘unable to cope’. Having trained locally and with aspirations to secure a consultant post in the same trust, he was acutely aware that calling the consultant overnight was not the ‘done thing’.

However, as Nirasha deteriorated, Alex's fear of inaction and emotional responsibility for death became more powerful motivators than institutional protocols or fear of retrospective critique. Clinicians of all levels are conceptually challenged by the idea of nonintervention (Borgstrom et al. 2020), which drove Alex to intervene. Gawande (2014) expressed this most succinctly, ‘ultimately, death comes, and few are good at knowing when to stop’ (p. 154). In this case, stopping meant not only accepting clinical futility but also risking a feeling of personal moral culpability, and Alex could not bear that weight.

Power/knowledge among junior doctors ought to be fostered through supported decision‐making during in‐hours shifts, where senior consultants are present to provide supervision and role modelling. However, the intense clinical workload meant that junior staff were routinely excluded from these critical learning moments. During Vidhya's conversation with Nirasha's family about her deterioration, the senior house officer was asked to complete discharge paperwork for another patient. At the same time, the registrar fielded unit referrals, leaving little opportunity to engage with the complexity of Nirasha's care. These structural interruptions fragmented the epistemic chain; junior doctors were physically present but discursively absent.

This absence was further compounded by visible disengagement from junior staff. On several ward rounds, junior clinicians were observed scrolling through social media, answering emails or attending to nonurgent ward tasks. This appeared to suggest both a coping strategy and a recognition of their marginalisation.

Their exclusion reflects not merely oversight but an a priori institutional logic that systematically positions junior doctors outside the production of social and clinical truth. Even when armed with plausible, evidence‐based claims, their ability to intervene is constrained by their exclusion from ‘practices that systematically form the objects of which they speak’ (Foucault 1969). As such, their power/knowledge is arrested: They cannot enact truth, cannot contribute meaningfully to care decisions and are left untrained in the discursive performances required to shape clinical reality.

## 7 Balancing Acts: Care in Tension

I picked up a neglected blood gas from the workbench and asked John, the respiratory consultant, ‘Is this important?’. It was, but no one had acted on it. The patient, Harry, had no notes and was only partially known to the team after shift change, so NARS was started urgently. The mask wouldn’t seal, and nurses tightened it again and again. ‘It’s horrible,’ John admitted, ‘but the machine needs it to work.’ As Harry deteriorated, specialist staff adjusted pressures. When he pulled at the mask, they gave sedation. Harry gave a series of blood gas samples from his wrist; each interpreted the same way—Harry was failing to recover. When John explained to the team it was clear Harry would not survive, the palliative care nurse stepped in, guiding his family through a peaceful withdrawal. Harry died that afternoon, quietly, mask removed, hand in hand with his son.

Harry was critically ill from the moment he was admitted, but his care was impacted by basic clinical errors (a blood gas left on a workbench, missing medical notes). In the absence of contextual knowledge, the clinical team defaulted to NARS, a pattern consistent with the moral imperative to intervene described earlier. However, once initiated, NARS did more than deliver oxygen; it began to structure care. Its outputs (blood gas results, leak rates and ventilation volumes) were treated with epistemic weight. This weight was granted through their interpretation by respiratory specialists, particularly ventilation nurses and consultants, whose power/knowledge conferred legitimacy on the machine's data.

In this way, the machine exerted agency: It demanded attention, drove decisions and began to act as a co‐author of care. Patients who became anxious or noncompliant were not managed because they were suffering, but because their distress disrupted the machine's function. Symptom control medications were often only deployed when agitation interfered with the machine's needs, demonstrating that patient welfare is subordinate to the machine's needs. What emerged was not just a reliance on NARS but a reorientation of clinical focus around its operational needs; an enactment of technology‐in‐practice (Orlikowski 2000), where the machine becomes a structuring force within the therapeutic encounter.

The machines' ability to wield power derives from their physical and conceptual properties. NARS machines cannot detect pain, fear or distress, but they can measure compliance and flow rates. These are the values that become narratively, clinically and epistemically privileged. Through accumulated experience, staff learn to associate machine compliance with survival, often recalling dramatic recoveries of previously moribund patients. As Orlikowski and Gash (1994) argue, the interpretation of the machine's agency develops through engagement, skill, training and previous experience. In this context, staff co‐construct a logic in which machine compliance becomes a proxy for hope and thus a rationale for persisting with burdensome treatment.

As Harry's condition worsened, the discourse around his prognosis became increasingly fractured. Specialist ventilation nurses, palliative care staff, and the medical team each offered divergent views about whether NARS should be continued. This tension gradually coalesced around Harry's visible distress. *‘I'm not sure we're doing the right think here’ John said to me after ward round. After seeing a few more patients John resolved, ‘Let's check his blood gas, if it's not improved, we stop’. It hadn't, and care transitioned towards palliation*.

With the machine's influence diminished by its failure to effect recovery, the specialist knowledge that had once governed its use no longer structured the discursive field. Power/knowledge ceased to circulate around respiratory expertise and instead consolidated around the palliative care team, who now provided the singular narrative guiding care. Although the palliative team often positioned themselves as advisory rather than authoritative, in cases like Harry's they effectively became decision‐makers, quietly and without formal declaration. Notes from his afternoon review simply read: “*under palliative care. Plan: (1) as per palliative*”. In these moments, the epistemic weighting of the machine fell away, replaced by a softer, singular dialogue between palliative nurses and families. Where the machine once generated complexity and contestation, palliation offered a form of narrative closure, allowing distress to be reframed as suffering, and suffering could now be acted upon.

In doing so, the palliative care team acted as death brokers, contributing to the social acceptability of death. A key component of this brokering was often, as shown in Harry's case, the removal of the mask and the avoidance of prolonged death. NARS treatment represents a liminal space, but it also becomes a form of social death. Inability to communicate, hear or be seen contributes to a declining capacity for social connection. As Seale (1998) writes, ‘Taking control over the manner and timing of one's own death… means the avoidance of a lingering social death’ (p. 172). Palliative care specialists frequently worked to restore this social connection in dying, encouraging small but meaningful acts, like Harry's son holding his hand, as a counterbalance to prior isolation. With the medical team's curative task now exhausted, the power/knowledge of the palliative team filled the vacuum, quietly subsuming control over care decisions and reconfiguring the end of life as a socially and morally coherent event.

## 8 Faith in the Machine

‘I think we’ve got one for you,’ the ward sister said as I arrived; it was June, a patient who had seemed to be recovering but had deteriorated overnight. Now on NARS, she lay unresponsive as a specialist nurse, Angie, took a blood gas. The NARS created a rhythmic rise and fall of her chest, but beyond this there were no obvious signs that June was still alive. ‘NIV for COPD is like magic,’ the nurse said; others had called it a deus ex machina, a literary device creating salvation at the last moment. But for June, the miracle never came, the blood gases were unchanged, and decisions deferred again until the next ‘scripture’ arrived. By the afternoon, even her son, with no medical training, had learned the central question: ‘What’s her carbon dioxide level?’

Timmermans (1999), in *Sudden Death and the Myth of CPR*, describes how resuscitative efforts become ritualised acts of faith in the face of clinical uncertainty. ‘Faith always seems to be involved in resuscitation’ (p. 54), he notes. NARS is not resuscitation, but it does occupy a similar cultural position; it is a last‐ditch intervention that symbolically and clinically resists death. In this study, staff (especially respiratory specialists) held a deep‐seated belief in the power of the NARS machine, choosing to initiate treatment even when the clinical likelihood of recovery was negligible. This faith, created by previous experiences of unexpected recoveries, often contributed to significant treatment burden and the medicalisation of dying for those who did not recover.

The language staff used revealed the extent of this belief. The machines always work, but ‘*some patients just don't get better’, as Angie put it.* Even when staff acknowledged poor prognoses, hope was sustained by the idea that the machine might still work. In June's case, for example, she exhibited several features indicating a poor outcome: reduced consciousness, severe acidosis and late‐onset acidosis. Her NIVO score was nine (indicating a 71% inpatient mortality rate) (Hartley et al. 2021). Yet Angie framed this in terms of possibility: ‘*Even if the NIVO score says 70% mortality, there's still a 30% chance of survival, you just never know*’. Clinical reasoning was shaped less by statistical likelihood and more by the ritualised logic of any chance being enough, and this was underpinned by a belief that the machine would work.

This conviction helped position NARS as the default approach to deterioration, reconfiguring the clinical gaze to centre the machine. Specialist knowledge about NARS settings and interpretation became epistemically privileged, as did the machine's own outputs (compliance metrics, pressure data, and above all, the blood gas). Once initiated, the machine began to act with agency within care. The ritual had begun.

Importantly, the machine's agency did not begin only at application; it extended retroactively into decision‐making. As in Harry's case, even when no clear indication existed, the team still initiated NARS while searching for a justification to do so. The machine's potential to provide salvation dominates the medical landscape. Latour (2004) characterises such nonhuman actors as possessing the capacity to ‘authorize, allow, afford, encourage, permit, suggest, influence, block, render possible, forbid’ (p. 226). NARS agency is deeply influential. Loud and insistent alarms fill the air if the mask is removed. Nurses are set to stay nearby the patient in case the machine calls for help. The protocol of care around NARS routes itself through the machine's outputs, especially the blood gas. Printed on small scrolls of curling paper, these tests became almost scripture to staff who held the faith of NARS.

Early in treatment, these blood gases are crucial to tuning machine settings and interpreting patient response, the only site of technical communion between human and nonhuman actors. However, even as patients deteriorated and interlocutors recognised the inevitability of death, they often deferred major decisions until another blood gas had been obtained. In June's case, despite consensus that she was dying, treatment was not de‐escalated until a final blood gas confirmed high carbon dioxide levels: The patient had failed to ‘let the machine work’. In other cases where death was recognised as inevitable, the blood gases had normalised; in these cases, it was taken that the machine had worked, but the patient had failed to get better. The meaning of death itself was now being narrated through the logic of machine outputs.

In this way, faith in the machine supplanted, or at least displaced, critical clinical judgement. A patient might appear clinically to be dying, but the blood gas was still sought, not for its practical utility but as a final opportunity to commune with the machine. The act of drawing a gas became less a diagnostic step than a moment of ritual consultation. The machine, through its outputs, was not only participating in decision‐making but also framing what kinds of decisions were imaginable. It created knowledge but also demanded that this knowledge be acted upon. In Foucauldian terms, the machine did not simply exist within a regime of power/knowledge; it began to generate and circulate that regime. Power circulated through the machine, allowing it to structure time, shape communication, and confer authority on those specialists who could interpret its voice.

## 9 Discussion

This ethnographic study explored the use of NARS in acute care settings, with particular attention to the sociological dimensions of decision‐making at the end of life. Drawing on Foucault's (1980) concept of power/knowledge and Orlikowski's (2000) theory of technology‐in‐practice, the study explores how clinical authority, truth production, and moral decision‐making are co‐constructed through discourse, institutional norms, and material technologies.

Across the fieldwork, the authority to declare a patient as dying, rather than critically ill, was not simply bestowed upon the most senior clinician. Instead, it was contingent on negotiations between the wider multidisciplinary team. Foucault's conceptualisation of power as circulating, relational, and embedded in systems of knowledge allows us to understand how this declaration was shaped by a distributed network of actors, including specialist staff, clinical documentation, institutional expectations, and the NARS machine itself. The ‘truth’ of dying emerged not as a clinical fact but as a product of discourse, often only stabilising when consensus was reached across the team.

The presence of the NARS machine itself played a constitutive role in shaping care decisions. As this study demonstrates, the machine functioned not only as a therapeutic tool but also as a nonhuman actor with agency, scripting clinical behaviour and producing epistemically privileged outputs (blood gases, pressures and compliance measures) that prioritised survival—even in the face of suffering. Orlikowski's notion of technologies‐in‐practice allows us to see how the machine's outputs became central to clinical narratives, producing routinised decision points and mediating communication with both team members and families.

The impact of epistemically weighted knowledge and technology can influence the relationship between care providers and patients. Mackintosh and Agarwal et al. (2022) examined the experience of obstetric patients accessing digital information, highlighting how clinical staff policed the boundaries of informational legitimacy by selectively validating or dismissing data and their sources. Similarly, in this study, staff curated the epistemic weight of machine‐generated outputs, explicitly demonstrating trust in the machine and endorsing its readings to patients and loved ones. This dynamic was evident in the consistent foregrounding of machine data (such as carbon dioxide levels) in discussions of care.

Epistemically weighted data produced by machines are, in fact, co‐produced by patients' bodies. Wood (2016) explored this dynamic in relation to the co‐production of images between patients and computed tomography (CT) scans, noting that patients are expected to conform to the needs of the CT machine (keeping their bladder full and rectum empty). The power/knowledge surrounding such technologies prioritises data acquisition, which, in turn, shapes the delivery of patient care. With regard to NARS, failure to meet the needs of the machine and behave as a ‘good patient’ can result in sedation or mask adjustments to enforce compliance.

Technological modifications to the mask interface can, and often do, improve treatment delivery. Actor‐network theory enables us to conceptualise technology not as passive equipment but as an active participant within care networks. It is open to adjustment, reconfiguration and strategic negotiation. In acute settings, where patients are severely unwell and less able to be educated or engaged, they often become relatively inflexible users of technology. By contrast, modifying the machine (tightening the mask, altering the interface) can maintain treatment delivery, though often at the expense of patient autonomy. Prout (1996) described a similar dynamic in relation to metered‐dose inhalers, highlighting how devices and users are mutually constituted in use. In this context, actor‐network theory helps us examine how patients and machines co‐adapt in ways that are socially and ethically consequential.

The power to declare patients were dying often circulated around the machines themselves, privileging staff with specialist knowledge of interpretation and management above more junior staff. Specialist staff's trust in the machine enhanced its power in decision‐making, as was demonstrated by Montague et al. (2010) in relation to medical machines in obstetrics. As a corollary, this privileging of specialist knowledge in decision‐making disengaged and disenfranchised more junior and generalist staff, which appeared to impact their training and development.

Because of the power/knowledge operating around and through the machines, other clinical management decisions became affixed and subservient to the ‘will of the machine’. This was most notable around the use of symptom control medications; patients were much more likely to receive symptom control medications if their symptoms interrupted the work of the machine. The NARS machine was unable to quantify patient comfort, and this, therefore, focused attention onto patient compliance.

One of the most striking findings of this study was the fracturing of clinical authority. The term ‘fracturing’ is used here to illustrate the disruption of the power/knowledge chain, which occurred when institutional expectations shifted from senior‐led care (during the day) to junior‐led care (at night). Senior consultants shaped the ‘official’ plan during the day, but junior doctors frequently enacted care overnight. At times, they acted in defiance of the plan, under emotional and moral duress. Rather than being empowered decision‐makers, these junior clinicians were frequently disempowered by daytime exclusion and then held accountable for the difficult decisions they were forced to make in isolation. The resulting moral labour was marked by anxiety, uncertainty and fear of inaction—a dynamic that deserves further critical attention within sociological analyses of healthcare hierarchies.

Often perceived ‘mistakes’ from junior staff overnight occurred because they lacked the authority to proclaim truth. However, despite widespread and intense criticism of these decisions, there was no formal, constructive feedback system. This poor training can lead to poor implementation of end‐of‐life care (Nevin et al. 2020), which was seen on multiple occasions in this study. In this context, deficiencies in training reflected a lack of observed practice opportunities with structured feedback on trainee performance. Although the consequence was absent or poor learning opportunities, the factors that produced these conditions were structural: high clinical workload, acute patient demand and shift patterns that fragmented continuity for junior staff.

This fracture in clinical authority is not simply a reflection of existing hospital hierarchies. Rather, the NARS machine itself participates in the production and destabilisation of those hierarchies. It privileges specialist knowledge, scripts clinical action and withholds authority from those without the appropriate epistemic capital. This dynamic is exemplified in the relationship between specialist nurses and registrar‐level doctors: Although registrars occupy a formally higher position within traditional medical hierarchies, specialist nurses often command greater authority in interpreting and acting on machine data.

Here, we see the convergence of Orlikowski's (2000) concept of technology‐in‐practice and Foucault's (1980) power/knowledge. The machine does not just reflect existing power hierarchies; it actively participates in reforming them. The fracture is not just institutional but technological and is now etched into the routines and expectations of care.

The impact of this technological reconfiguration of authority can be further seen in the concept of ‘blood gas scripture’—the small scrolls of paper containing blood gas results. Clinicians and families interpreted these as gospel. In some cases, this ‘scripture’ was so foregrounded in decision‐making that clinical ‘truth’ was not derived from a holistic assessment of the patient but from the interpretation of machine‐generated data. The privileging of machine outputs, which we have referred to as their epistemic weight, reaffirms the roles of knowledge, power and technology in shaping how death is recognised and managed. In doing so, NARS machines disrupt a longstanding medical hierarchy in which doctors alone held the authority to declare death and dying.

These findings align with Pols (2017), who asserts that technologies’ introduction to the clinical space necessitates active engagement from clinicians. Clinicians must tame, interpret or even resist technologies' influence within care practices. The values that emerge from such practices cannot be determined in advance; they are realised through use, improvisation and institutional constraint. NARS does not merely support life or delay death but reshapes the moral landscape of care itself.

Once decisions had been made to transfer to palliative care, the machines no longer held a position within decision‐making. Simultaneously, the specialist care of patients often transitioned away from medical consultants to palliative care specialist teams. Although palliative care teams were often perceived as advisory, their involvement frequently resulted in a reorientation of care and a consolidation of authority around mask withdrawal and symptom control. This transfer of moral responsibility, while relieving clinical tension, creates questions about institutionalised boundaries of care and the uneven distribution of ethical labour.

The findings of this study contribute to ongoing sociological debates around the relational and distributed nature of clinical decision‐making, the impact of medical technology on those decisions and the moral ambiguity of end‐of‐life care. They show that the power to act, to decide or to withhold treatment is not the possession of any one actor but emerges from a web of interactions in which discourse and technology are mutually constitutive.

This study focuses on the interactions between staff, patients and technology in a ward‐based setting. However, there are clear analogies between the experiences of patients in this context and those receiving care in intensive care units (ITUs). Seymour (2000) describes the active construction of a patient's death as a ‘negotiation over the meaning of medical–technical data and of the technical treatments given [a patient] [and] the less easily articulated but clear recognition of inevitable bodily dying.’ As in this study, meaning is co‐constructed through epistemically weighted machine outputs (haemofiltration, ventilator parameters or, in our case, blood gases and pressures). In both contexts, the power/knowledge chain privileges those with institutional and discursive authority. The capacity to interpret machine data and shape clinical narratives remains concentrated among senior or specialist staff, whereas junior clinicians are excluded from meaning‐making despite being tasked with enacting care.

Pols (2017) argues technologies in healthcare should not be treated as a singular category, nor assumed to exert uniform effects across contexts. Each device brings particular nuance, constraints and interpretive demands that must be attended to in contextualised ways. Although there are clear analogies between NARS and other technologies (such as haemofiltration or mechanical ventilation), caution is warranted when transferring insights between them. Attending closely to the particulars of each technology allows us to more accurately understand how clinical practices are shaped and influenced by specific technologies.

Where translational learning may be more applicable is in the moral impact technologies have on clinical staff. Bandini (2020) describes the moral distress experienced by ICU clinicians when navigating the ‘buffet’ of technological options available to prolong life. The availability of such interventions creates a perceived ethical obligation to offer them, even when their benefits are uncertain or their burdens significant. As in our study, clinicians must shoulder the emotional and moral labour of asking: Just because we can intervene, does it mean we should?

## 10 Implications and Future Directions

This research provides new insights into the sociological literature on end‐of‐life care by demonstrating how noninvasive ventilation technologies do not simply support or delay dying but actively participate in the production of clinical truths about who is dying and when. Although prior work has examined how medical technologies shape trajectories of death (e.g., Timmermans 1999, 2005; Seale 1998), this paper uniquely shows how the epistemic authority of machine outputs, especially blood gas readings, can supplant embodied clinical judgement and delay palliation. Moreover, by foregrounding the role of junior clinicians and the temporal fracturing of institutional authority, the study explored how care is shaped not only by clinical hierarchy but also by infrastructural rhythms that leave critical decisions in the hands of disempowered actors. In doing so, the paper extends current understandings of technological agency and moral labour in acute care, offering a novel account of how machines, institutions and professionals co‐produce the boundaries of life and death.

This study offers four key implications. First, it suggests that interventions aimed at improving end‐of‐life care must attend not only to communication or policy but also to the material infrastructures, such as the NARS machine, that shape what kinds of care are conceivable and visible.

Second, it highlights the need for greater support and inclusion of junior and nonspecialist staff in decision‐making processes. Their moral labour is real, consequential and often unacknowledged. Inclusion in daytime decision‐making would provide opportunities for them to wield power/knowledge more effectively, equipping them to make difficult clinical judgements at night. Without this exposure, they face crises in isolation, lacking the discursive authority to act. Addressing this imbalance would help ensure that epistemic authority circulates across the full 24‐h cycle of hospital care, rather than collapsing at night in the absence of senior staff.

Third, it calls for further research into how ‘truths’ about dying are stabilised in clinical settings, not just through clinical observation or expert knowledge but through institutional logics, material scripts and the epistemic authority of machines.

Finally, this study underscores the continued need for sociological engagement with the conceptualisation of nonintervention in dying and critically ill patients. NARS amplifies the dynamics identified by Kaufman (2015), in which biomedical technologies sustain both the possibility and the expectation of continued treatment, even when prospects of recovery are low. Our findings contribute to the wider shift called for by Cohn et al. (2025), who argue for reframing medicine's purpose, ‘away from medicine's main purpose being to protect life and forestall death, to one in which its role is to continually engage with qualities of living, such that dying is no longer conceptualised as medicine's failure but as a vantage point from which to acknowledge the changing biological and social conditions everyone will experience.’ Although this study was situated in a single healthcare institution and focused specifically on NARS, its findings are likely to resonate more broadly across healthcare systems where end‐of‐life care is technologically mediated. The construction of clinical truth, the negotiation of professional authority and the moral complexity of end‐of‐life care are not unique to NARS, the NHS or the United Kingdom; they are features of many acute care settings internationally. Similar sociotechnical dynamics may be observed in other interventions (such as intensive care, dialysis or artificial nutrition and hydration) which rely on epistemically weighted, objective measures of efficacy but often lack equivalent means of assessing patient comfort. However, the configuration of these dynamics, including who interprets machine outputs, how palliative transitions are negotiated and how junior staff are supported, may vary considerably depending on local policy, training structures and cultural framings of death and dying. Future comparative research could usefully explore how these processes manifest across different institutional and national contexts.

## Author Contributions

**David Wenzel:** conceptualization, data curation, formal analysis, writing – original draft, writing – review and editing. **Jennifer Creese:** conceptualization, supervision, writing – review and editing. **Eleanor Wilson:** conceptualization, supervision, writing – review and editing. **Michael Jones:** validation, writing – review and editing. **Christina Faull:** conceptualization, supervision, writing – review and editing.

## Data Availability

The data that support the findings of this study are available upon request from the corresponding author. The data are not publicly available due to privacy or ethical restrictions.
